# Recent Development of Back-Contacted Single-Crystal Perovskite Solar Cells

**DOI:** 10.3390/ma19112415

**Published:** 2026-06-05

**Authors:** Xiao Cheng

**Affiliations:** School of Materials Science and Engineering, Shandong University, Jinan 250061, China; chengxiao@sdu.edu.cn

**Keywords:** back-contacted perovskite solar cells, stability, single-crystal perovskite, single-crystal thin films

## Abstract

The efficiency of perovskite solar cells has increased to a certified value of 27% over the past decade, benefiting from the superior properties of metal halide perovskite materials. However, their long-term operational stability is still far inferior to that of commercial crystalline silicon solar cells. A key source of this instability is field-driven ion migration in vertical architectures, along with the consequent degradation at the absorber–electrode interfaces. Compared with the widely investigated vertical structures, back-contacted (BC) perovskite solar cells—wherein both electrodes are positioned on the same side of the absorber—offer a unique route to suppress interfacial ion migration and thereby enhance long-term device stability. These advantages are especially pronounced when combined with single-crystal perovskites, which possess low charge trap densities, long carrier diffusion lengths, and high bulk ion migration barriers. Unfortunately, only a handful of research groups have participated in the development of single-crystal BC perovskite solar cells; thus, the advancement of this area lags far behind that of its vertical counterpart. Therefore, a review that discusses the recent developments and challenges of single-crystal BC perovskite solar cells is urgently required to provide guidelines for this emerging field. In this progress report, we first introduce the main growth methods of single-crystal wafers compatible with BC architectures, followed by an outline of the developmental history of BC perovskite solar cells. Finally, the core bottlenecks facing single-crystal BC devices and corresponding optimization strategies are discussed in detail.

## 1. Introduction

Driven by the increasing global energy demand and the urgent need to reduce carbon emissions, the development of efficient and low-cost renewable energy technologies has become critically important. Among various renewable energy sources, photovoltaic technology is regarded as one of the most promising routes for clean electricity generation. Currently, crystalline silicon solar cells dominate the photovoltaic market owing to their high efficiency and long-term stability, while thin film technologies, such as CdTe and CIGS, provide alternative commercial routes with reduced material consumption. In addition, III–V nanowire solar cells have attracted attention as next-generation high-efficiency photovoltaic systems because dense nanowire arrays can enhance light absorption and reduce active material consumption. For example, Prete and Lovergine discussed the potential of III–V nanowire solar cells based on intermediate-band absorbers [1], while Cretì et al. reported enhanced optical absorption in GaAs/AlGaAs core–shell nanowires [2]. However, these technologies still face challenges, such as high fabrication costs, complex epitaxial growth, material scarcity, or limited large-scale manufacturability. In this context, metal halide perovskites have emerged as promising photovoltaic materials due to their high absorption coefficients, tunable bandgaps, long carrier diffusion lengths, low-temperature solution processability, and rapidly increasing power conversion efficiency [3,4].

Organic–inorganic hybrid halide perovskites have attracted widespread attention in the optoelectronic field because of their outstanding optoelectronic properties, including high absorption coefficients [5], high carrier mobilities [6], long carrier diffusion lengths [7], and tunable bandgaps [8]. Particularly in the field of photovoltaics, the power conversion efficiency (PCE) of perovskite solar cells has rapidly increased from the initial 3.8% to above 27% [9,10], outpacing all other solution-processed photovoltaic materials while gradually approaching the efficiency level of crystalline silicon solar cells. From the perspective of commercialization, however, stability remains the key bottleneck that limits further development, and a substantial gap still exists relative to crystalline silicon. One important origin of the rapid performance decay of perovskite solar cells lies in material and electrode degradation induced by halide ion migration [11,12,13]. State-of-the-art perovskite solar cells generally adopt a vertical sandwich architecture [14], in which the direction of the built-in electric field is aligned with that of ion migration, thereby providing a direct driving force for ionic motion [15]. In polycrystalline perovskite thin films, halide ion migration mainly occurs at the buried surface, the grain boundaries, and the perovskite/metal electrode interface due to the pervasive grain boundaries [16,17,18]. In contrast, the ion migration pathways in perovskite single crystals are greatly reduced and are concentrated primarily at the interfaces between the perovskites and the electrodes.

At present, mainstream crystalline silicon solar cells are mainly based on TOPCon (tunnel oxide passivated contact) and HJT (heterojunction) architectures, and the efficiency of commercial modules has reached 25% [19]. In comparison, back-contacted (BC) architecture has not yet been industrialized on a large scale, but it has already delivered the highest efficiencies in silicon photovoltaics. In 2025, LONGi reported a hybrid back-contacted crystalline silicon solar cell with an efficiency of 27.81%, further narrowing the gap between silicon cell performance and its theoretical limit [20]. Inspired by this design concept, researchers have recently begun to introduce BC architectures into perovskite solar cells. In BC perovskite devices, the positive and negative electrodes are located on the same side of the perovskite crystal or polycrystalline film, and charges must be transported and collected in both the vertical and lateral directions, which places much more stringent requirements on carrier transport properties. Polycrystalline perovskite films, owing to their abundant grain boundaries, suffer from limited carrier diffusion lengths and numerous ion migration pathways, both of which are unfavorable for device stability [21]. In contrast, single-crystal perovskites, with carrier diffusion lengths on the order of several hundred micrometers and higher bulk ion migration barriers, are ideal absorber materials for high-efficiency and stable BC solar cells [22].

Conventional vertical PSCs typically rely on transparent conductive oxide electrodes, such as indium tin oxide (ITO) and fluorine-doped tin oxide (FTO), which represent one of the major cost contributors in PSC fabrication [23,24]. Cost analyses based on life cycle assessment, sustainable manufacturing analysis, and recycling oriented evaluation suggest that TCO substrates can account for approximately 40–60% of the total device cost of PSCs [25,26,27]. Wang et al. reported that, for typical n–i–p PSCs, the TCO, HTL, and metal electrode account for approximately 30%, 53%, and 16% of the total raw material cost, respectively. The high cost of the HTL is mainly associated with expensive organic hole transport materials, such as spiro-OMeTAD, while the cost of the metal electrode is related to the use of noble metals, such as Au and Ag [28]. From a module-level perspective, Larini et al. predicted that TCO-coated glass accounts for approximately 56% of the total cost of perovskite modules, highlighting the dominant contribution of TCO substrates to module cost [29]. Because BC architectures eliminate the need for these expensive transparent electrodes, they are expected to reduce fabrication costs substantially.

In addition, ITO electrodes introduce extra optical losses. Van Eerden et al. found through optical analyses of planar perovskite solar cells that ITO and its adjacent functional layers generate pronounced interfacial reflection and parasitic absorption, thereby reducing the effective photon flux reaching the perovskite absorber [30]. Jacobs et al. further showed that absorption by the ITO film itself weakens its transmittance, especially in the long-wavelength region, where optical losses become more pronounced [31]. Therefore, by removing the transparent conductive electrode and combining the device with an effective antireflection coating, the BC architecture is expected to enhance light harvesting and further improve the short-circuit current density (*J*sc) and PCE. Beyond cost reduction and optical management, the BC configuration may also address a degradation pathway that is unique and particularly critical in perovskite photovoltaics: electric field-driven ion migration. Compared with silicon cells, BC architectures offer an additional and distinctive advantage in perovskite photovoltaics, namely the suppression of ion migration. Because the direction of the built-in electric field is approximately orthogonal to the direction of interfacial halide ion migration, the driving force for interfacial ion migration is weakened, which should in principle be beneficial for improving operational stability, especially when coupled with the intrinsically weak bulk ion migration of perovskite single crystals. On the other hand, the carrier transport pathways in BC and vertical devices are also fundamentally different. In vertical architectures, carriers must be transported toward the bottom electrode, whereas partial delamination of the perovskite layer often occurs during device operation and creates voids at the perovskite/ITO interface, thereby reducing interfacial charge extraction and accelerating performance decay [32]. In BC devices, by contrast, carriers no longer need to be transported to a bottom electrode, avoiding performance deterioration caused by local delamination of the perovskite layer and thus, in principle, mitigating device aging. Li et al. found that, under otherwise identical single-crystal compositions, interfacial modifiers, and electrode materials, BC perovskite solar cells exhibited superior operational and thermal stability compared with their vertical counterparts [33].

Beyond material cost and device performance, the practical viability of PSCs also depends on their life cycle energy input and operational lifetime. Energy payback time (EPBT) is generally defined as the time required for a photovoltaic module to generate the same amount of energy that was consumed during its production. A shorter EPBT indicates that the photovoltaic technology can recover its embodied energy more rapidly, reflecting higher energy return efficiency and better environmental sustainability. Gong et al. performed a cradle-to-grave life cycle assessment of two representative solution-processed perovskite solar modules, including an FTO/Au/mesoporous TiO_2_-based module and an ITO/Ag/ZnO-based module. Their results showed that perovskite solar modules exhibited shorter EPBT values than many existing photovoltaic technologies, with the ZnO-based module showing an average EPBT of approximately 0.2 years [34]. Furthermore, Tian et al. conducted a life cycle assessment of high-performance perovskite tandem solar cells. The all-perovskite tandem cell adopted a monolithic two-terminal architecture, consisting of a wide bandgap perovskite top cell and a narrow bandgap Pb–Sn perovskite bottom cell connected in series. A representative device stack can be described as ITO/PTAA/perovskite (wide bandgap)/C_60_/SnO_X_/perovskite (narrow bandgap)/C_60_/BCP/Cu. Their analysis showed that this all-perovskite tandem configuration exhibited an EPBT of approximately 0.35 years, further demonstrating the potential of perovskite photovoltaics for rapid energy return [35]. For single-crystal BC PSCs, the elimination of transparent conductive oxide electrodes and the potential for improved operational stability may help reduce manufacturing energy input and extend the effective energy generation lifetime, thereby further improving their energy return.

Research on BC perovskite solar cells has a history of nearly one decade, but overall progress has been rather slow and still lags far behind that of vertical perovskite devices. Dong et al. first fabricated a BC perovskite solar cell based on a bulk MAPbI_3_ single crystal, but its PCE was only 1.88% under 0.25 sun illumination [36]. It was not until 2020 that Song et al. increased the efficiency of BC perovskite solar cells to above 10% by optimizing the interfacial energy level alignment between the MAPbI_3_ wafers and the electrodes while simultaneously repairing interfacial defects [37]. More recently, Han et al. reduced the densities of point, line, and planar defects in perovskite wafers through compositional engineering and improved carrier transport properties, pushing the record for efficiency in this field beyond 17% [38]. Although this value still falls well short of that of vertical perovskite solar cells, the performance has already shown a certain degree of application competitiveness when the elimination of costly transparent electrodes is taken into account.

From the viewpoint of carrier transport, perovskite wafers with micrometer- or even nanometer-scale thicknesses are favorable for shortening the vertical transport distance and reducing recombination losses, thereby enabling high-efficiency BC perovskite solar cells. Recent studies have shown that, compared with bulk single crystals, perovskite wafers still display clear deficiencies in crystal quality, defect control, and carrier transport, which in turn point to directions for further performance improvement [39,40]. In addition, reports on BC solar cells remain limited, and their interfacial modification mechanisms and carrier transport kinetics have yet to be elucidated in depth. Meanwhile, the ion migration and degradation mechanisms in these devices are still not fully understood, which further constrains stability enhancement. A systematic review of the current status of this field and the key challenges it faces is therefore of great significance. In this review, we first summarize the growth methods for perovskite wafers that are suitable for BC architectures and analyze the merits and drawbacks of these methods in detail. We then outline the development history of BC perovskite solar cells before finally discussing the key factors governing their performance and future directions for this field.

## 2. Methods for Growing Perovskite Wafers

**Figure 1:**
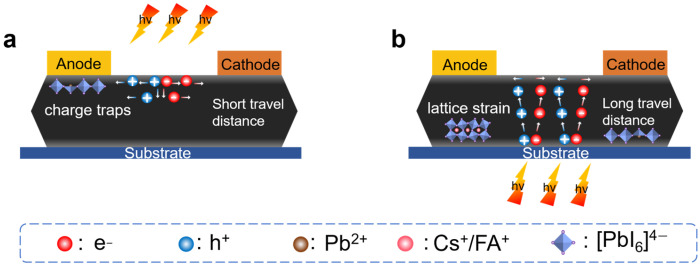
Schematic diagram of the carrier generation and transport process in a single-crystal BC solar cell when light is incident from (**a**) the back side with electrodes and (**b**) the front side without electrodes [41]. Copyright 2026, ELSEVIER.

Figure 1 illustrates the device architecture and carrier transport pathways of BC perovskite solar cells. When light is incident from the back side with electrodes, photogenerated carriers are mainly distributed at the region approximately 500 nm underneath the top crystal surface, considering the penetration depth of visible light. On one hand, the carriers need to travel laterally across the electrode spacing. On the other hand, the vertical diffusion of carriers will extend the traveling distance before being collected by both electrodes (Figure 1a). When light is incident from the front side without electrodes, these carriers must travel across both the crystal thickness in the vertical direction and the electrode spacing in the lateral direction before they can be collected. After reaching the electrode side, electrons are selectively extracted by the electron transport layer and then collected by the cathode, while holes are extracted by the hole transport layer and collected by the anode (Figure 1b). Meanwhile, the electron and hole transport layers block opposite carriers, thereby suppressing interfacial recombination during charge collection [41]. From the vertical transport perspective, a thinner perovskite crystal is more favorable for suppressing carrier recombination, provided that efficient light absorption is maintained. Therefore, the growth of high-quality, low-defect perovskite wafers with excellent carrier transport properties is the foundation for constructing high-efficiency BC perovskite solar cells. At present, the methods most relevant to such devices mainly include surface tension-assisted growth, the space-confined method, geometrically confined lateral single-crystal growth, and epitaxial growth.

### 2.1. Surface Tension-Assisted Growth

**Figure 2:**
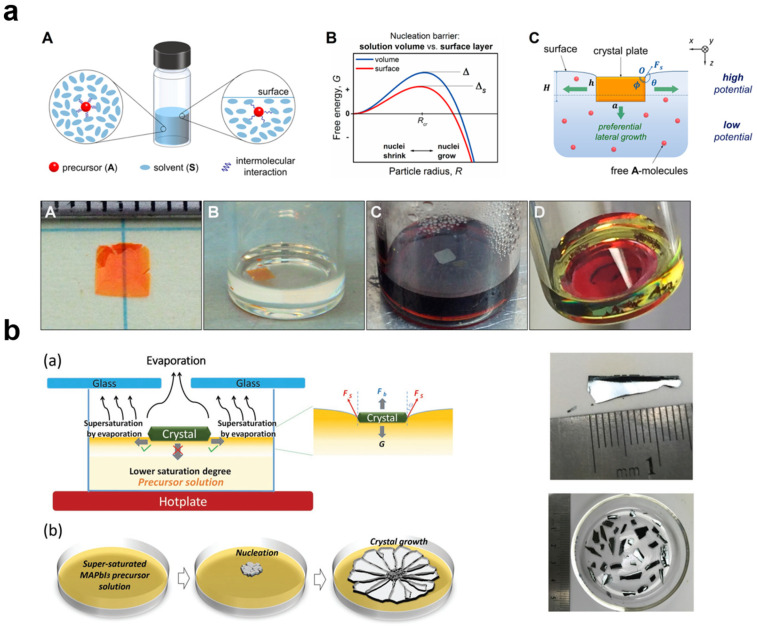
(**a**) Schematic illustration of the mechanism for growing perovskite single-crystal thin films using the surface tension-assisted growth method, with photographs of the obtained MAPbBr_3_, MAPbI_3_, and MASnBr_3_ single-crystal thin films [42]. Copyright 2017, American Chemical Society. (**b**) Scheme of the crystal growth mechanism and a photograph of a 1.5 cm long piece of MAPbI_3_ single-crystal wafer grown from a 5 cm diameter container [43]. Copyright 2019, Wiley-VCH.

Surface tension-assisted growth is one of the earliest methods used to grow perovskite wafers. As shown in Figure 2, solvent evaporation acts as the driving force, and crystal nucleation and growth are realized at the solution surface by regulating the supersaturation and interfacial interactions. The formation of the crystal usually proceeds in two steps. During nucleation, solvent evaporation enriches the surface layer first, allowing it to reach supersaturation before the bulk solution and thereby inducing nucleation at the liquid surface. During the subsequent lateral growth stage, the increased solution density and surface tension support the floating crystal. Continuous surface evaporation sustains supersaturation near the liquid surface and drives preferential lateral growth, ultimately yielding micrometer-thick perovskite wafers. As shown in Figure 2a, Zhumekenov et al. first applied this method to halide perovskites and successfully obtained MAPbI_3_, MAPbBr_3_, MASnBr_3_, and MASnI_3_ single-crystal thin films with areas of approximately 1 cm^2^ and thicknesses of 5–10 μm, demonstrating a certain degree of generality for wafer growth [42]. As shown in Figure 2b, Liu et al. further extended the solution surface growth concept to MAPbI_3_ single-crystal wafers. By exploiting evaporation induced supersaturation at the air–liquid interface, nucleation was preferentially triggered on the surface of the precursor solution, followed by rapid lateral growth supported by interfacial forces and continuous precursor supply from the solution. This strategy enabled the formation of wafer-scale MAPbI_3_ single-crystals with a thickness of approximately 38 μm within 30 min, demonstrating the feasibility of fast surface-mediated growth for ultrathin perovskite single-crystal wafers [43].

This method features a relatively simple process, low equipment requirements, and facile control of the growth behavior through adjustment of the precursor concentration, solvent evaporation rate, and liquid surface environment, enabling the rapid production of large single-crystal sheets [44]. Because nucleation occurs locally at the liquid surface and lateral growth is sustained by surface tension, the resulting crystals are often difficult to keep intact and continuous, which limits crystal integrity [45]. In addition, the strong coupling between nucleation, solvent evaporation, and solute transport at the liquid surface usually leads to relatively thick crystals with insufficient thickness uniformity and controllability [46]. To obtain wafers with sufficiently large lateral dimensions, the nucleation density must generally be reduced, which lowers the number of single crystals available for device fabrication in each growth cycle and limits material throughput [47]. Overall, although this method can produce micrometer-thick perovskite wafers, it still faces challenges in crystal integrity, thickness control, and scalable reproducible production [48].

### 2.2. Space-Confined Method

**Figure 3:**
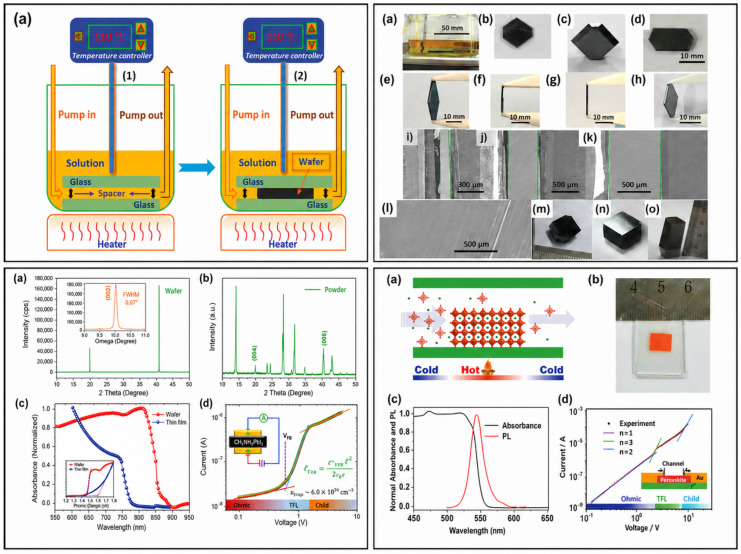
(**a**) Schematic illustration of the space-confined method used to grow perovskite single-crystal thin films; two flat glass slides are separated by two spacers to form a microreactor. (**b**) Photograph of the microreactor; photographs and cross-sectional SEM images of MAPbI_3_ single-crystal thin films with different thickness and shapes; and photographs of MAPbI_3_ single crystals grown using conventional processes. (**c**) XRD, rocking curve, absorption, and space-charge-limited current characterizations of the single-crystal thin films. (**a**–**c**) Reproduced with permission [50]. Copyright 2016, Wiley-VCH. (**d**) Schematic illustration of the growth of MAPbBr_3_ single-crystal thin films using the space-confined method; photoluminescence, absorption, and space-charge-limited current characterizations, as well as a photograph, of the as-grown MAPbBr_3_ single-crystal thin films [53]; reproduced with permission.

To overcome the limited thickness controllability and crystal integrity associated with surface tension-assisted growth, the space-confined method has become the most widely used route for preparing perovskite wafers. It generally combines the inverse temperature crystallization mechanism with physical space confinement to regulate crystal growth. After the perovskite precursors are dissolved in organic solvents, such as DMF, DMSO, and GBL, the inverse solubility of the material is exploited; that is, the solubility decreases with increasing temperature. By increasing the temperature, the solution is gradually driven to saturation, thereby triggering crystal nucleation and growth [49]. Meanwhile, the precursor solution is sandwiched between two parallel glass substrates to form a slit-like confined space, which restricts growth in the vertical direction; the spacing between the two substrates determines the wafer thickness. As shown in Figure 3a, Liu et al. first developed the space-confined method by constructing a geometry-defined dynamic flow microreactor consisting of two glass plates and spacers, enabling controlled growth of MAPbI_3_ wafers with thicknesses adjustable from 150 to 1440 μm (Figure 3b). XRD and rocking curve full width at half maximum (FWHM) analyses demonstrated the high crystallinity of the resulting wafers, while space-charge-limited current (SCLC) measurements revealed a trap density as low as 6 × 10^8^ cm^−3^ [50] (Figure 3c). Rao et al. subsequently extended this method to the MAPbBr_3_ system and obtained wafers with an area of 120 cm^2^ and a thickness of approximately 400 μm, as well as wafers with an area of 48 mm^2^ and a thickness of approximately 16 μm [51,52]. Chen et al. further extended the method to MAPbCl_3_ and, with the assistance of external pressure, achieved thickness control of MAPbBr_3_ single crystals from the nanometer scale to the micrometer scale. The obtained MAPbBr_3_ thin single crystals showed a sharp near-band-edge PL emission, indicating good optical quality. In addition, SCLC analysis gave a low trap density of approximately 3.1 × 10^10^ cm^−3^ and a hole mobility of 42 ± 9 cm^2^ V^−1^ s^−1^ [53] (Figure 3d).

**Figure 4:**
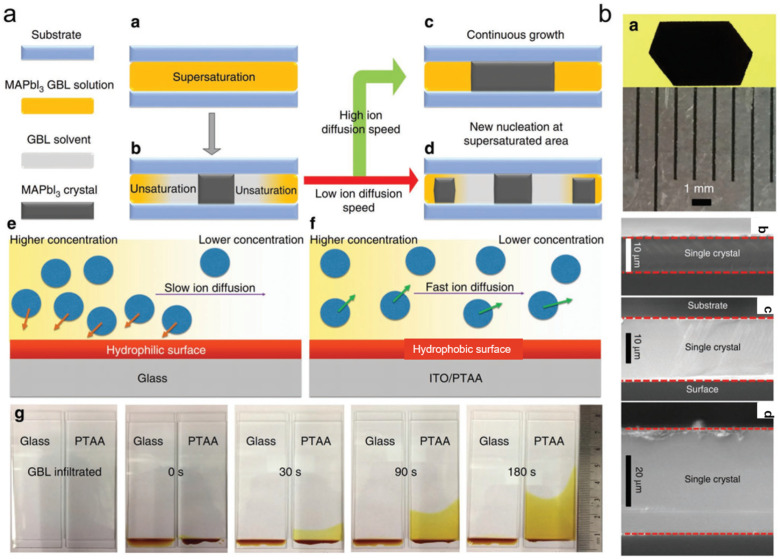
(**a**) Growth mechanism of the hydrophobic interface-confined lateral crystal growth method. Schematic illustrations and photographs of the ion diffusion process in the limited space using hydrophilic and hydrophobic substrates. (**b**) Photographs and cross-sectional SEM images of MAPbI_3_ single-crystal thin films with different thickness. Reproduced with permission [55]. Copyright 2017, Springer Nature.

The space-confined method still faces several challenges in the growth of iodide-based perovskite crystals, including slow solute diffusion, random nucleation positions, and limited lateral dimensions. For example, although Zhao et al. obtained MAPbI_3_ single crystals with thicknesses of 3–10 μm on FTO/TiO_2_ substrates, the morphology was irregular and the lateral size was only several hundred micrometers [54]. To address this issue, Chen et al. developed a hydrophobic interface-confined lateral growth method for MAPbI_3_ single-crystal wafers. As illustrated in Figure 4a, the conventional confined growth process suffers from slow ion diffusion in the narrow gap. By introducing a hydrophobic interface, the precursor solution can diffuse more rapidly within the confined space, leading to a more uniform solute supply and continuous lateral growth of the single crystal. As shown in Figure 4b, this strategy enabled the formation of millimeter-scale MAPbI_3_ single-crystal wafers with smooth surfaces and controllable thicknesses of 10–40 μm, as confirmed by optical images and cross-sectional SEM characterization [55]. Huang et al. further employed hydrophobic and porous PDMS to regulate slow solvent evaporation and solute diffusion, thereby preparing mixed-cation mixed-halide single crystals with thicknesses of 10–50 μm and lateral sizes from 500 μm to 2 mm [56]. Compared with surface tension-assisted growth, the space-confined method enables more effective thickness control and improved crystal integrity, making it more suitable for the fabrication of BC solar cells.

The space-confined method is reasonably universal and can be applied to a variety of perovskite compositions. It exhibits distinct advantages in wafer thickness control and surface flatness. Nevertheless, several intrinsic drawbacks remain. First, during crystal growth, the residual solution in the confined space can redissolve the organic components at the single-crystal surface, while the subsequent slow cooling process may further induce volatilization of organic species, thereby introducing surface damage and defects [57]. Second, because the crystal remains under rigid constraint from the substrate during both growth and cooling, and because pronounced differences in thermal expansion coefficients exist between perovskites and common substrates, such as glass or silicon, the internal stress arising from thermal mismatch can readily induce lattice distortion, local cracking, and even microcracks in the crystal bulk and at the buried interface, significantly degrading crystal quality [58]. In addition, the space-confined method places stringent demands on the uniformity of the local growth environment [59]. Stable and reproducible high-quality wafers are easier to obtain in a small area; however, during scale-up, the uniformity of the confined gap becomes difficult to maintain, often leading to spatial variations in growth rate and crystallinity, ultimately impairing wafer continuity and uniformity [60,61]. Thus, although the space-confined method is presently the mainstream route for preparing high-quality single-crystal thin films, it still faces key problems, including a high density of surface defects, incomplete stress release during growth, and insufficient uniformity over large areas.

### 2.3. Geometrically Confined Lateral Single-Crystal Growth

**Figure 5:**
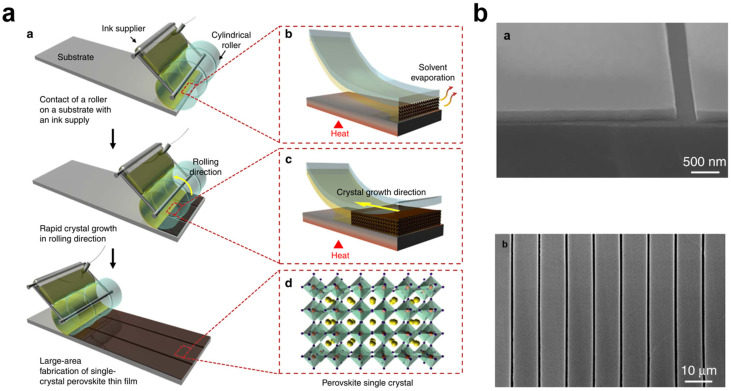
(**a**) Schematic of the manufacturing procedure for single-crystal perovskite thin films using geometrically confined lateral crystal growth (GC-LCG) with a rolling mold. (**b**) Scanning electron microscopic (SEM) image of single-crystal perovskite patterned thin film, consisting of 10 mm wide strips with 400 nm wide spacing, and cross-sectional SEM image of 200 nm thick perovskite single-crystal strips, showing sharp edges and smooth morphology [62]. Copyright 2017, Springer Nature.

The geometrically confined lateral single-crystal growth method can produce perovskite single-crystal films over a large area. Although the films are discontinuous, they can still satisfy the requirements for BC solar-cell fabrication. In this method, a preheated substrate with a temperature above the solvent boiling point is brought into contact with a patterned mold loaded with precursor ink. Rapid solvent evaporation generates a local highly supersaturated environment and induces crystal nucleation. At the same time, the geometric channels in the mold restrict crystal growth in the vertical direction and guide directional transport of precursor ions along the lateral direction, allowing the dominant nuclei to continue growing while suppressing randomly oriented crystallites, thus ultimately achieving oriented nucleation and lateral expansion of single crystals. Lee et al. first reported this method in 2017 [62]. As shown in Figure 5a, they wrapped a flexible mold with groove structures around a roller and continuously transferred the precursor ink onto a preheated substrate during rolling. In this way, they realized controllable lateral growth of perovskite single-crystal films and obtained patterned wafer-scale films with an area of 3 × 3 inches and a thickness of approximately 200 nm. The as-grown films comprised single-crystal stripes about 10 μm wide, with linear gaps of roughly 400 nm remaining between adjacent stripes (Figure 5b). This method shows clear advantages for large-area fabrication while also offering controllable growth direction, a high degree of film patterning, and strong potential for continuous production. In particular, its roll-printing-like process is favorable for extension to high-throughput manufacturing, while parameters such as groove size and rolling speed provide a process basis for regulating film thickness and morphology.

Its drawbacks are also quite prominent. First, the method generally relies on relatively high growth temperatures and rapid solvent evaporation, which can perturb local supersaturation and stoichiometric balance during crystallization and thereby introduce additional defects that impair crystal integrity [62]. Second, the process window is narrow and highly sensitive to parameters such as temperature, evaporation rate, mold geometry, and the confined gap. During scale-up, excessive nucleation sites and incomplete connection of single-crystal stripes can readily occur, resulting in deteriorated film continuity and uniformity [63]. Third, the upper and lower confining interfaces and the groove edge regions may introduce additional residual stress and local stress concentration, further inducing structural distortion and lowering the quality of single-crystal films [64]. Therefore, although geometrically confined lateral single-crystal growth opens a new avenue for large-area single-crystal film preparation, further optimization is still required in crystal quality control, process stability, and defect suppression [65].

### 2.4. Epitaxial Growth

**Figure 6:**
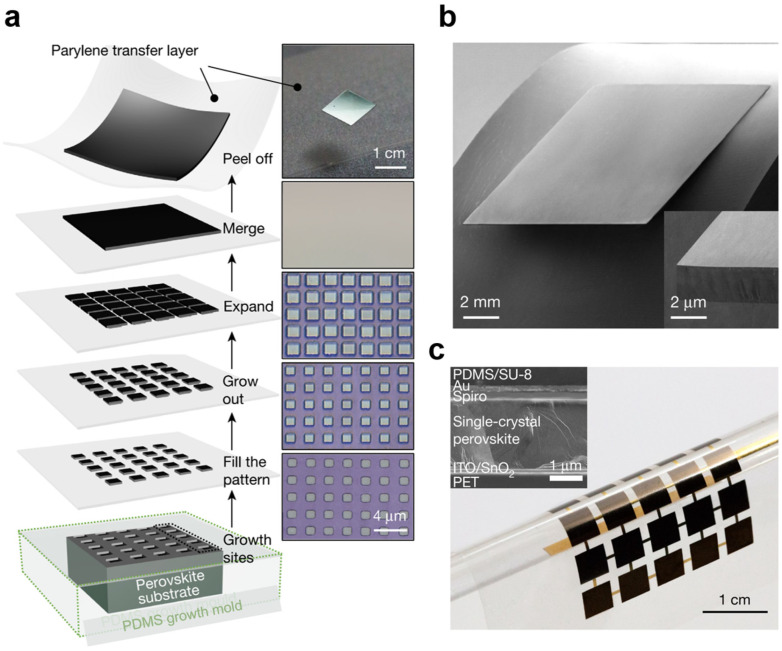
(**a**) Schematics (left) and corresponding optical images (right) showing the solution-based epitaxial growth, merging, and transferring processes of single-crystal perovskite thin films; the bottom four optical images share the same scale bar, 4 μm. (**b**) SEM images showing a single-crystal MAPbI_3_ thin film with dimensions of about 1 cm × 1 cm × 2 μm on a bent PDMS substrate; inset shows a magnified cross-section of the thin film without grain boundaries. (**c**) Optical image showing an array of flexible single-crystal photovoltaic islands with a total working area of 6.25 cm^2^; inset shows a cross-sectional SEM image of the single-crystal perovskite photovoltaic device [68]. Copyright 2020, Springer Nature.

Epitaxial growth is a highly mature method in the semiconductor industry for preparing single-crystal thin films. Epitaxial growth uses a single-crystal template to guide the oriented nucleation of perovskites on the substrate surface. Under controlled supersaturation, the epitaxial domains expand laterally and coalesce into a continuous film, thereby transferring the crystallographic orientation of the template to the perovskite layer and enabling large-area single-crystal film fabrication. Chen et al. prepared continuous CsPbBr_3_ single-crystal thin films on SrTiO_3_(100) substrates by vapor-phase epitaxy. In this method, incommensurate lattice matching between CsPbBr_3_ and the oxide perovskite SrTiO_3_ induced heteroepitaxial nucleation, while regulation of the nucleation density suppressed the tendency toward Volmer–Weber island growth. As a result, continuous single-crystal thin films with tunable micrometer-scale thicknesses were obtained on 5 mm × 10 mm substrates, with a representative film thickness of approximately 7 μm [66]. Yang et al. further reported a solution epitaxy method for preparing CsPbBr_3_ thin single-crystals. By regulating the nucleation and lateral growth of the precursor solution on the substrate surface, high-quality thin single-crystals could be obtained under mild conditions, with the crystal thickness reduced to approximately 1.6 μm and the lateral size reaching the millimeter scale, providing a feasible route for low-temperature solution processing of thin halide perovskite single-crystals [67]. Lei et al. reported template-assisted epitaxial growth of large-area perovskite wafers. As shown in Figure 6a, perovskite crystals were first grown from predefined growth sites in a patterned PDMS mold. The crystals then expanded laterally, merged into a continuous single-crystal film, and were finally peeled off with the assistance of a Parylene transfer layer. Figure 6b shows the obtained large-area single-crystal wafer with a smooth and continuous morphology, while the SEM image confirms its micrometer-scale thickness [68]. More importantly, the transferable wafer could be integrated with flexible substrates, as demonstrated by the flexible single-crystal perovskite device shown in Figure 6c.

## 3. Development History of Single-Crystal Back-Contacted Solar Cells

Research on BC perovskite solar cells began with bulk crystals, and the corresponding devices initially exhibited a PCE of only 1.88%. Subsequent efforts shifted toward thickness-controlled wafers in order to reduce the vertical carrier transport distance. Through crystal composition regulation, defect passivation, and interface engineering, the PCE of BC solar cells has now surpassed 17%, demonstrating a certain degree of competitiveness.

### 3.1. Back-Contacted Solar Cells Based on Bulk Single Crystals

**Figure 7:**
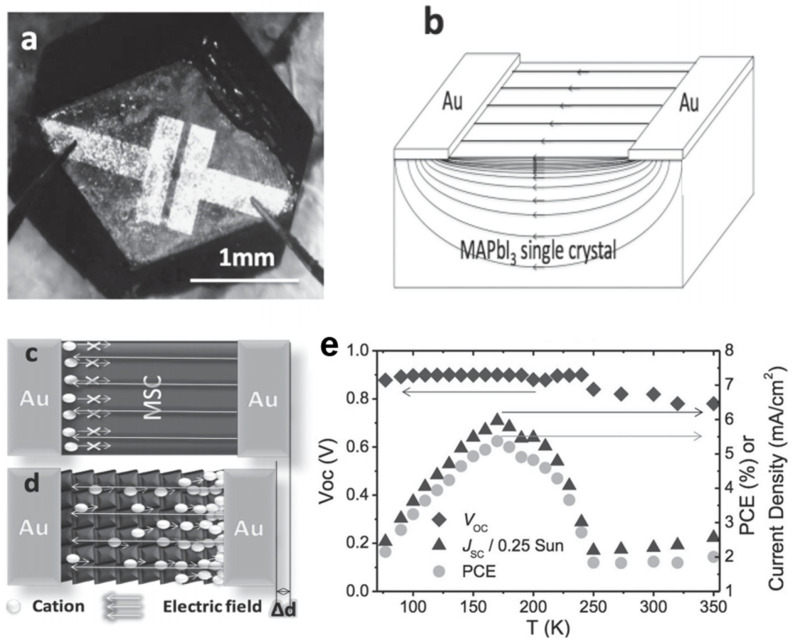
(**a**) Image of MAPbI_3_ BC structure single-crystal device. (**b**) Illustration of the poling electric field distribution in top surface and cross-section of a lateral single-crystal device. (**c**,**d**) Scheme of electromechanical strain-generated grain boundaries and ion migration in MAPbI_3_ single crystals. (**e**) Photocurrents of the MAPbI_3_ single-crystal and polycrystalline thin films with similar electrode spacing of 50 µm under 25 mW cm^−2^ illumination at room temperature [36]. Copyright 2016, Wiley-VCH.

In 2016, Dong et al. prepared bulk MAPbI_3_ single crystals by the acid solution-cooling method and used high-voltage-induced ion migration to form a p-i-n junction, thereby promoting directional carrier transport. As shown in Figure 7a, the obtained MAPbI_3_ bulk single crystal had a millimeter-scale lateral size. The device structure was Au/MAPbI_3_/Au, in which two Au electrodes were deposited on the same side of the single crystal to form a lateral back-contacted configuration (Figure 7b). Under high-voltage poling, mobile ions migrated along the electric field direction, leading to the formation of a built-in p-i-n junction inside the MAPbI_3_ single crystal, as illustrated in Figure 7c,d. The solar cell delivered a PCE of 1.88% under 0.25 sun illumination, significantly outperforming polycrystalline BC devices with the same structure and thus providing an initial proof of concept for BC architectures in perovskite solar cells (Figure 7e) [36].

### 3.2. Back-Contacted Solar Cells Based on Perovskite Wafers

**Figure 8:**
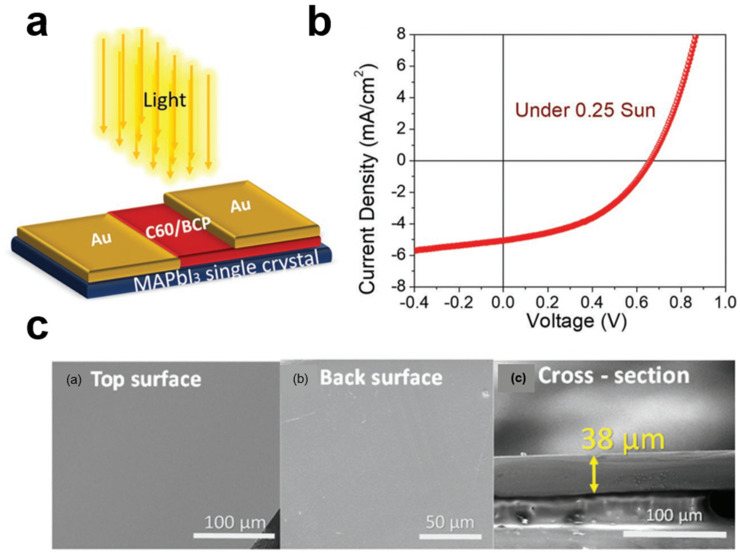
(**a**) Scheme of MAPbI_3_ single-crystal wafer lateral structure solar cell device. (**b**) *J*–*V* curve of our best co-planar lateral MAPbI_3_ single-crystal solar cell device with PCE of 5.9%. (**c**) SEM images of top surface, back surface, and cross-section of MAPbI_3_ single-crystal wafer [43]. Copyright 2019, Wiley-VCH.

Although bulk single crystals provided the first proof of concept for BC perovskite photovoltaics, their large thickness inevitably prolonged the vertical transport distance and intensified bulk recombination. This limitation motivated the transition from bulk crystals to micrometer-thick perovskite wafers. In 2019, Liu et al. fabricated BC solar cells with an Au/MAPbI_3_/C_60_/BCP/Au structure using micrometer-thick MAPbI_3_ wafers grown via the surface tension-assisted method. As shown in Figure 8a, C_60_/BCP was introduced between the MAPbI_3_ single crystal and one Au electrode as the electron transport layer, forming a lateral back-contacted device structure. By introducing this electron transport layer at the crystal/cathode interface, they avoided grain boundary and dislocation defects associated with polarization effects. The obtained MAPbI_3_ wafer showed smooth top and back surfaces, and the SEM image confirmed a thickness of approximately 38 μm (Figure 8c). Under 0.25 sun illumination, the device achieved a PCE of 5.88%, as shown by the *J*–*V* curve in Figure 8b, marking an important step in the development of BC single-crystal solar cells [43].

**Figure 9:**
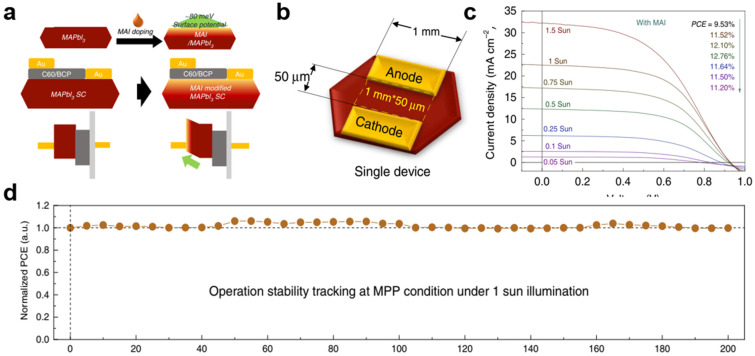
(**a**) Schematic diagrams of device structures and energy levels for SC-PSCs, with and without MAI treatment. (**b**) Structure of a regular single device with an area of 50 μm × 1 mm. (**c**) Light intensity (0.0005–1.5 sun) dependence of *V*_OC_ curves; *J*–*V* curves of different light intensity with MAI treatment. (**d**) Long-term stability under continuous output at MPP condition (1 sun) [37]. Copyright 2020, Springer Nature.

The space-confined method yielded intact crystals while reducing the thickness of perovskite wafers to around 10 μm. Song et al. were the first to construct BC solar cells with an Au/MAPbI_3_/C_60_/BCP/Au structure based on MAPbI_3_ wafers grown by the space-confined method. In general, iodide-based perovskite crystals grown from solution exhibit a deficiency of organic components at the surface, resulting in a high density of surface defects. Song et al. introduced an ultrathin methylammonium iodide (MAI) layer onto the perovskite wafer surface, effectively replenishing the organic species lost through high-temperature volatilization and reducing surface defects, such as iodide vacancies. In addition, the work function of the Au electrode differed greatly from that of the perovskite, leading to interfacial energy level mismatch and voltage loss. As shown in Figure 9a, after MAI treatment, the average surface potential of the single crystal increased by about 80 meV, leading to improved energy level matching between the single-crystal surface and the Au electrode, reduced interfacial recombination, and enhanced charge collection. As shown in Figure 9b, the electrode spacing was 1 mm × 50 μm. As a result, a PCE of 11.52% was achieved under 1 sun illumination at room temperature, representing the highest efficiency in the field at that time (Figure 9c). They also found that the BC solar cells exhibited excellent operational stability, with no decay during maximum power point (MPP) tracking for 200 h (Figure 9d) [37].

**Figure 10:**
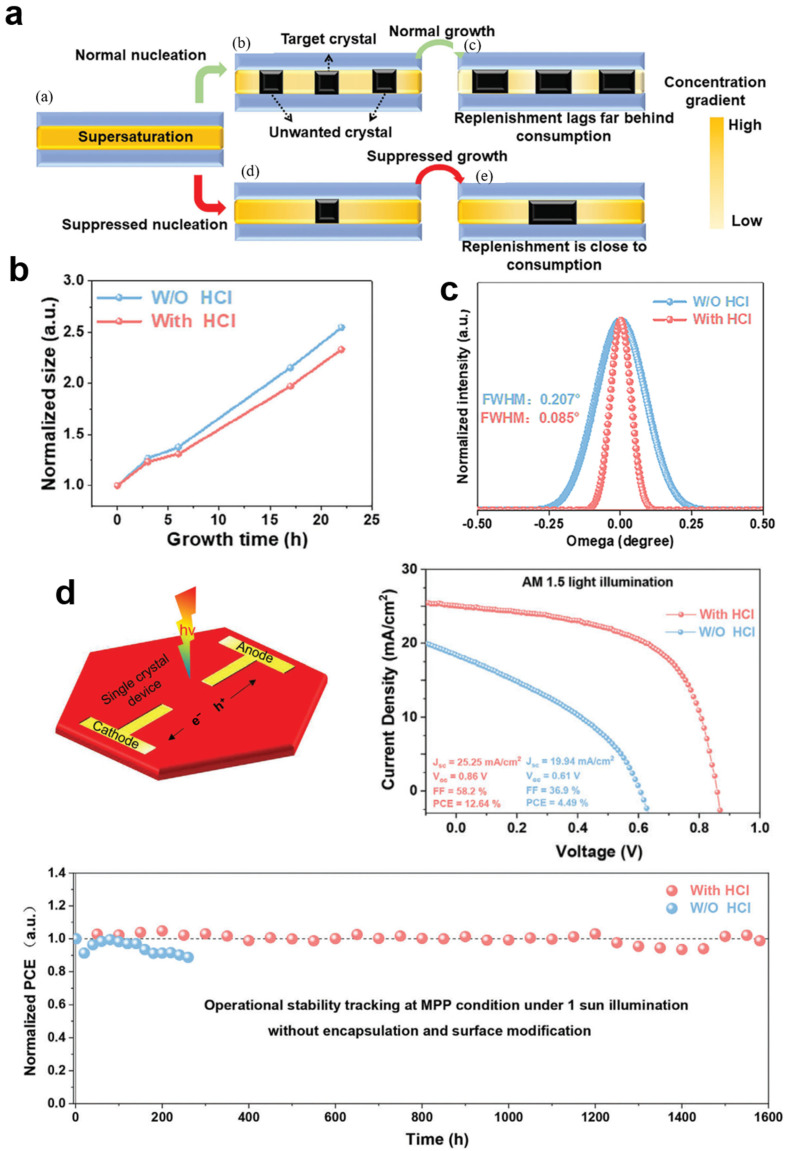
(**a**) Schematic illustration of growth of thin single crystals by the space-confined method. (**b**) Normalized size of single crystals, with and without HCl-modulated growth, as a function of growth time. (**c**) High-resolution rocking curves of the (220) diffraction of single crystals, with and without HCl-modulated growth. (**d**) Schematic illustration of BC structure of single-crystal solar cells, *J*–*V* curves, and operational stability of BC single-crystal PSCs, with and without HCl-modulated crystal growth, under AM 1.5 light illumination [33]. Copyright 2024, Wiley-VCH.

In addition to surface defects, the crystal quality of perovskite wafers grown by the space-confined method is clearly inferior to that of bulk crystals, resulting in a higher density of bulk defects. To address this issue, Li et al. introduced HCl gas into the crystal-growth solution to regulate nucleation and growth. By suppressing unwanted nucleation events (Figure 10a), this strategy ensured that the replenishment of solute was close to its consumption during crystal growth, minimizing concentration gradients and allowing for the preparation of larger and higher-quality crystals. The effect of HCl on suppressing nucleation and growth was confirmed by the normalized crystal size over time (Figure 10b), showing a slower growth rate for crystals grown with HCl. Improved crystallinity was further verified by XRD and rocking curves (Figure 10c), where the full width at half maximum (FWHM) of the (110) peak decreased from 0.207° (without HCl) to 0.085° (with HCl). As a result, the crystallinity and carrier transport properties of MAPbI_3_ wafers were significantly improved, and the power conversion efficiency (PCE) of BC solar cells was raised to 12.64%, compared to only 4.48% for devices without HCl treatment. They further compared ion migration in BC and vertical architectures and found markedly weaker metal/perovskite interfacial ion migration in BC devices because the migration direction was nearly orthogonal to the electric field, greatly reducing the driving force for ion migration. Consequently, the BC solar cells exhibited much better operational stability, showing no efficiency decay after 1200 h of MPP tracking under 1 sun illumination. (Figure 10d) [33].

**Figure 11:**
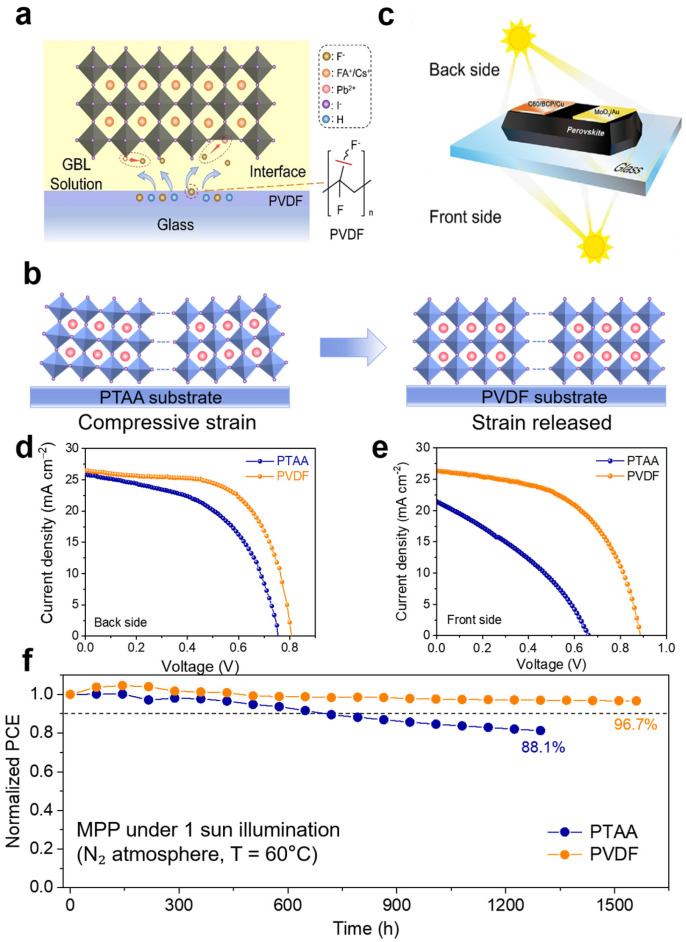
(**a**) Schematic diagram of the fluorine atoms released by the decomposition of PVDF at the crystal/substrate interface during crystal growth process. (**b**) Schematic diagram of the lattice arrangement of perovskite single crystals on PTAA and PVDF substrates. (**c**) Schematic structure of the single-crystal back-contacted devices. (**d**) *J*–*V* curves of the devices based on PTAA and PVDF substrates when light is incident from (**d**) the back side and (**e**) the front side; (**f**) Operational stability of back-contacted Cs_0.05_FA_0.95_PbI_3_ single-crystal PSCs when light is incident from the back side [41]. Copyright 2026, ELSEVIER.

Beyond the crystal surface and the interior interfacial stress between the crystal and the substrate is another factor that limits device performance. After crystal growth, the perovskite must be cooled to room temperature, and the pronounced difference in thermal expansion coefficients between the glass substrate and the perovskite introduces interfacial stress. To address this problem, Liu et al. modified the glass substrate surface with poly(vinylidene fluoride) (PVDF), whose thermal expansion coefficient is close to that of the perovskite. Compared with the traditional PTAA substrate that causes compressive strain in the perovskite lattice, the relatively low elastic modulus of PVDF enables effective release of crystal growth stress through cooperative interfacial deformation, thereby reducing defect density and yielding higher-quality single-crystal wafers (Figure 11b). In addition, C-F bonds in PVDF can partially break in a γ-butyrolactone (GBL) environment, releasing free fluoride ions that coordinate with exposed Pb ions and fill iodide vacancies, thereby further reducing surface defects (Figure 11a). Through the synergistic effect of stress release and defect passivation, both lateral carrier transport and charge collection efficiency were enhanced, as evidenced by the improved *J*–*V* performance measured from both the front and back sides of the BC devices (Figure 11c–e). The PVDF-modified devices exhibited significantly higher performance than those with PTAA substrates, resulting in a PCE of 13.37% under 1 sun illumination, while the device maintained its performance and was unchanged, retaining 96.7% of its initial efficiency after 1560 h of MPP tracking under 1 sun illumination at 60 °C in an N_2_ atmosphere (Figure 11f) [41].

Because the PVDF-modified BC devices exhibited efficient charge collection under both front and back illumination, this work also revealed the potential of BC architectures for bifacial photovoltaic operation. On this basis, Liu et al. further found that the BC architecture also holds promise for bifacial photovoltaic operation. In contrast to conventional front-contacted PSCs, where front electrodes inevitably introduce optical shading and parasitic absorption, BC PSCs relocate both electron and hole collecting electrodes to the rear side of the perovskite absorber. As a result, the front surface can be fully exposed to incident light, which is beneficial for light harvesting under front side illumination and also provides a structural basis for bifacial power generation. Based on the PVDF modified single-crystal BC device, Liu et al. achieved a front side PCE of 12.80%, close to the back side performance and corresponding to a bifaciality factor of 0.96. When additional reflected light of 0.2 and 0.5 sun was introduced, the device output increased to 124% and 136% of its initial value, respectively, demonstrating the potential of BC PSCs for simultaneous utilization of direct and reflected light. Nevertheless, the interdigitated back-contacted electrodes still cause non-negligible optical loss under back illumination. After considering the electrode shading effect, the effective bifaciality factor decreased from 0.96 to 0.80.

**Figure 12:**
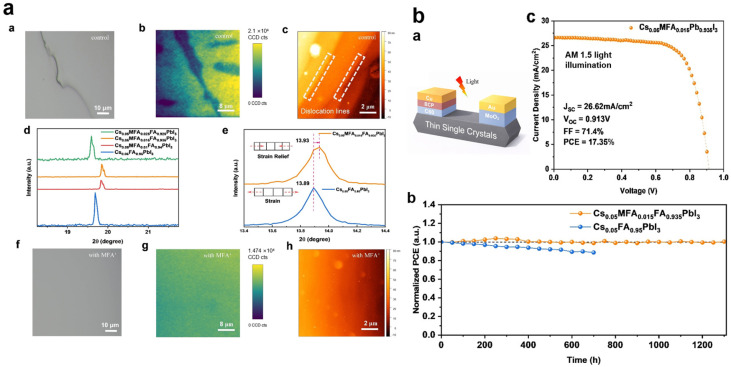
(**a**) Optical microscopy, PL intensity mapping, magnified (110) plane XRD patterns, and AFM topography of wrinkle regions in Cs_0.05_FA_0.95_PbI_3_ and Cs_0.05_MFA_x_FA_0.95−x_PbI_3_ (x = 0.01, 0.015, 0.025) thin single crystals. (**b**) Device structure of the back-contacted single-crystal solar cells, *J*–*V* curves of the champion Cs_0.05_MFA_0.015_FA_0.935_PbI_3_ devices, and the operational stability of the two back-contacted single-crystal solar cells [38]. Copyright 2026, Wiley-VCH.

However, although interfacial stress regulation and surface defect passivation can further improve device performance, the efficiency of single-crystal BC solar cells still remains clearly below that of vertical single-crystal devices. One key limitation is that the crystals still contain multidimensional structural defects, including point, line, and planar defects, all of which jointly deteriorate carrier transport and aggravate nonradiative recombination (Figure 12a). To address this bottleneck, Han et al. proposed a strategy for the synergistic suppression of multidimensional defects. By introducing MFA^+^ molecules into Cs_0.05_FA_0.95_PbI_3_ crystals, they strengthened the interaction between A-site cations and iodide ions, thereby effectively suppressing the formation of iodide vacancies while relieving tensile microstrain (Figure 12a) and eliminating line and planar defects, such as dislocations and surface wrinkles (Figure 12a). The resulting high-quality crystals exhibited an electron diffusion length of about 400 μm, indicating a marked improvement in lateral carrier transport. Ultimately, the BC solar cell fabricated from this crystal achieved a PCE of 17.35% (Figure 12b), representing a significant advance over previously reported BC PSCs. Moreover, no obvious performance decay was observed after 1350 h of continuous illumination (Figure 12b), demonstrating excellent operational stability [38].

At present, the highest efficiencies and best stabilities in BC solar cells have been achieved with perovskite wafers. Nevertheless, their relatively poor crystal quality and carrier transport properties still limit further performance gains. In particular, the rocking curve FWHM values of wafers obtained by the space-confined method are typically broadened by one to two orders of magnitude compared with those of bulk single crystals, indicating substantial room for crystal quality optimization [69,70,71,72,73,74,75]. In addition, current perovskite wafers—especially those used for BC device fabrication—are too small to meet application requirements. Epitaxial growth can provide large-area wafers more rapidly, but it remains unexplored in the field of BC solar cells.

### 3.3. Back-Contacted Solar Cells Based on Nanowire Arrays

**Figure 13:**
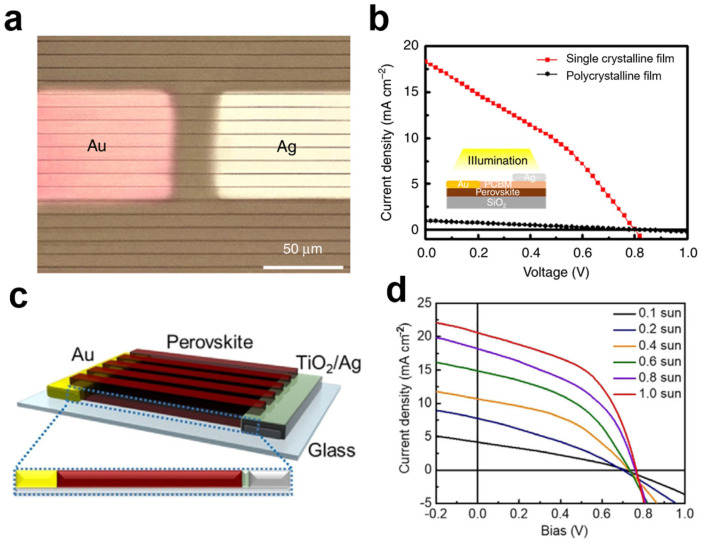
(**a**) Optical microscopic image of a single-crystal perovskite lateral perovskite solar cell with metal electrodes; light brown and dark brown lines indicate perovskite single crystals and spaces, respectively. (**b**) *J*–*V* curves of single-crystalline and polycrystalline thin film BC perovskite solar cells; inset shows the device structure of a lateral perovskite solar cell and the direction of illumination [62]. (**a**,**b**) Copyright 2017, Springer Nature. (**c**) Schematic illustration of a BC single-crystal perovskite device. (**d**) *J*–*V* characteristics of lateral single-crystal perovskite solar cells under illuminations of 0.1–1.0 sun [76]. (**c**,**d**) Copyright 2020, Wiley-VCH.

Geometrically confined lateral single-crystal growth can produce large-area perovskite nanowire arrays. Because the resulting films are discontinuous, they cannot be used to build vertical devices, but BC solar cells can still be fabricated by arranging the electrode array perpendicular to the direction of the nanowire array. Lee et al. constructed BC solar cells based on MAPbI_3_ nanowire arrays with an Au/MAPbI_3_/PCBM/Ag structure, in which the spacing between the Au and Ag electrodes was 33 μm (Figure 13a). Before testing, an electric poling process at 0.39 V μm^−1^ for 60 s was required to induce the formation of a lateral p-i-n junction within the perovskite layer. Under 1 sun illumination at room temperature, the devices achieved a PCE of 4.83%, and the *J*–*V* performance showed a clear superiority over polycrystalline film-based counterparts (Figure 13b) [62]. Considering the strong reactivity between Ag electrodes and perovskites, Park et al. inserted a TiO_2_ electron transport layer between the Ag electrode and the perovskite, while also employing asymmetric Au/Ag electrodes to strengthen the lateral built-in electric field (Figure 13c). Combined with poling-induced formation of an equivalent p-i-n junction, this strategy improved charge separation and collection. In addition, by optimizing the electrode spacing to a smaller scale, the series’ resistance and recombination loss during lateral transport were significantly reduced, ultimately yielding a PCE of 7.43% under 1 sun illumination, as verified by the *J*–*V* characteristics measured under different light intensities (0.1–1.0 sun) (Figure 13d) [76]. The nanowire arrays were only several hundred nanometers thick, so the vertical carrier transport distance was greatly reduced compared with that in the perovskite wafers discussed above. Nevertheless, the efficiencies of the corresponding BC solar cells still remain below 10%, indicating that the density of charge defects in the nanowire arrays was much higher than that in perovskite wafers. On the one hand, this is likely due to the increased specific surface area; on the other hand, the method relies on relatively high temperatures, which promote loss of organic components and introduce a large number of vacancy defects. Although this method has produced large-area array films, BC devices with large active areas remain to be explored.

## 4. Core Bottlenecks and Optimization Pathways for Single-Crystal Back-Contacted Solar Cells

Taken together, the evolution of single-crystal BC solar cells indicates that the focus of research has gradually shifted from early morphology control and electrode structure design toward deeper physical issues, including interfacial energy level matching, stress regulation, and intrinsic defect repair. Although the record efficiency has now exceeded 17% through the use of functional interlayers, surface passivation, and stress-buffering strategies, the performance potential of the BC architecture remains far from fully realized when compared with vertical single-crystal devices and polycrystalline thin film devices. On this basis, this section analyzes the technical challenges faced by present single-crystal BC solar cells from three core dimensions—lateral charge dynamics regulation, crystallization uniformity control, and long-term operational stability—and discusses frontier optimization pathways toward high-performance devices.

### 4.1. Efficiency Bottleneck: Charge Collection and Recombination Loss

The performance of BC solar cells is governed by the kinetic balance among carrier transport, geometric spacing, and recombination loss. Because photogenerated carriers must complete long-range lateral migration on the micrometer scale (typically tens of micrometers), together with a certain vertical transport distance and before they can be captured by the electrodes, the PCE is extraordinarily sensitive to the carrier diffusion length of the material. The underlying limitations can be broadly discussed in terms of growth stress and surface defect-induced restrictions on intrinsic transport. Zhao et al. systematically revealed the existence and origin of residual lattice strain in hybrid perovskite films by combining out-of-plane/in-plane X-ray diffraction, scraped powder reference samples, and in situ temperature-dependent XRD measurements. They found that perovskite films fabricated by conventional thermal annealing processes were generally strained, which originated from the large mismatch in thermal expansion coefficients between the perovskite layer and the ITO/glass substrate during the cooling process. As a result, the constrained perovskite films exhibited in-plane tensile strain and out-of-plane compressive strain. More importantly, such residual strain was found to be difficult to fully release by post-annealing once the perovskite film had formed on the substrate, highlighting the intrinsic nature of substrate-induced mechanical constraint in solution-processed perovskite films [77]. Kim et al. further demonstrated the significance of strain management in α-FAPbI3-based perovskites. By introducing Cs and methylenediammonium (MDA^2+^) into the perovskite lattice, they effectively reduced lattice strain, suppressed defect formation, and decreased the Urbach energy, leading to a certified power conversion efficiency exceeding 25%. These results indicate that lattice strain is not merely a structural perturbation but is closely associated with defect formation, energetic disorder, and photovoltaic performance [78].

The influence of residual stress on the optoelectronic properties of perovskites can be mainly understood from two aspects. On the one hand, stress-induced lattice distortion, dislocations, and microcracks can generate nonradiative recombination centers, which deteriorate carrier lifetime and transport. On the other hand, residual stress can reduce the activation energy for ion migration, thereby accelerating halide migration and perovskite decomposition. To directly clarify this mechanism, Zhao et al. tuned the strain state of MAPbI_3_ films by bending flexible substrates and compared the stability of films with different strain levels under continuous illumination. It was found that films with larger residual strain decomposed more rapidly into PbI_2_. Temperature-dependent conductivity measurements further showed that the activation energy for ion migration decreased with increasing strain, providing direct evidence that strain-assisted ion migration is responsible for the accelerated degradation of perovskite films [77]. More recently, Shen et al. showed that periodic lattice strain generated under day/night cycling can induce deep trap accumulation and chemical degradation in perovskite solar cells. They further found that the ion motion activation energy decreased from 0.25 to 0.13 eV after repeated cycling, indicating that strain evolution under operational light/thermal cycling can aggravate ion migration and accelerate device degradation [79]. Consistently, Kim et al. showed that strain release could prolong carrier lifetime, decrease defect density, and enable unencapsulated devices to retain more than 80% of their initial efficiency after aging at 85 °C in the dark for 1300 h [78]. Therefore, for back-contacted single-crystal perovskite solar cells, stress management is not only critical for maintaining crystal integrity but also essential for suppressing recombination losses and improving long-term stability during lateral carrier transport.

While residual stress mainly affects the lattice integrity and ion migration tendency of the crystal, surface defects impose a more direct constraint on carrier extraction, especially in BC devices where carriers are collected along near-surface lateral pathways. Wu et al. distinguished the surface and bulk recombination kinetics of MAPbBr_3_ and MAPbI_3_ single crystals by using one photon and two photon optical excitation methods. For MAPbBr_3_ single crystals, the bulk recombination lifetime was approximately 34 ns, whereas the surface lifetime was shortened to nearly 1 ns. The surface recombination velocity was estimated to be around 6.7 × 10^3^ cm s^−1^, and the surface trap density reached approximately 6.0 × 10^17^ cm^−3^, which was about two orders of magnitude higher than the bulk trap density of 5.8 × 10^15^ cm^−3^. Correspondingly, the diffusion length of surface-excited species was only 130–160 nm, much shorter than the bulk value of 2.6–4.3 μm [80]. These results clearly indicate that the low defect advantage of single-crystal perovskites mainly originates from the bulk, while the surface remains a defect-rich region that promotes charge trapping and trap-assisted recombination. This issue becomes particularly important in back-contacted single-crystal perovskite solar cells, where photogenerated carriers need to travel laterally through the crystal surface or near-surface region before being collected by the electrodes. Therefore, surface traps are located directly on the carrier transport pathway and can strongly limit carrier lifetime, diffusion length, and charge collection efficiency.

Guo et al. investigated MAPbI_3_ thin single-crystal solar cells and pointed out that bare Pb^2+^-related surface defects can induce severe surface nonradiative recombination and ion migration. By modifying the single-crystal surface with 3-mercaptopropyl(dimethoxy)methylsilane (MDMS), the sulfur atom of the molecule coordinated with the exposed Pb^2+^ sites, thereby reducing the surface defect density and suppressing recombination. As a result, the MAPbI_3_ single-crystal devices achieved an efficiency of 22.2%, together with enhanced moisture and reverse bias stability [81]. Liu et al. further demonstrated the dominant role of deficient crystal surfaces in Cs_0.05_FA_0.95_PbI_3_ single-crystal solar cells. Through optimized polishing engineering, the deteriorated surface was removed to obtain a more stoichiometric Pb/I surface composition, iodide vacancy concentration was reduced, ion migration activation energy was increased, and nonradiative recombination was suppressed. The resulting devices delivered an efficiency of 23.1%, and the T85 lifetime increased to 1150 h after the recycling process. More importantly, the repeated recovery of degraded devices by removing the deteriorated surface verified that device degradation in single-crystal perovskite solar cells is mainly governed by the crystal surface, while the effects of the bulk and buried interface are relatively weak [82].

#### Optimization Pathway: Surface Passivation and Interfacial Energy Level Engineering

**Figure 14:**
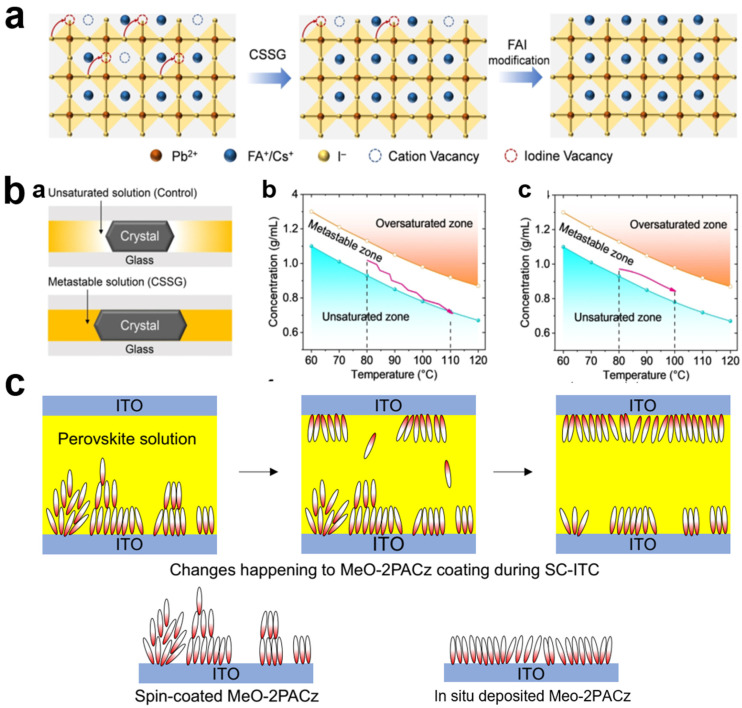
(**a**) Schematic diagram of hierarchical elimination strategy of surface iodide vacancies in perovskite single crystals. (**b**) Schematic diagram of single-crystal growth based on control and CSSG strategies, as well as the relationship between concentration and growth temperature for different regions in the crystal growth model using control and CSSG strategies [83]. (**a**,**b**) Copyright 2025 American Chemical Society. (**c**) Schematic illustrations of the in situ MeO-2PACz deposition process. Source data for the plots are provided as a source data file [84]. Copyright 2020, Springer Nature.

To repair defects and optimize transport, researchers have developed a variety of passivation strategies. To address the deficiency of iodide at single-crystal surfaces, Li et al. proposed a “stepwise surface defect management” strategy (Figure 14a). By dynamically replenishing the confined space with fresh precursor solution during growth, they precisely locked the crystal growth environment within a metastable region (Figure 14b), effectively maintaining stoichiometric balance throughout the growth process and eliminating defects in the crystal interior and in the near-surface region. On this basis, subsequent surface modification with organic ammonium salts (FAI) enabled comprehensive repair from the micrometer-scale subsurface region to the atomic-scale surface [83]. Yeddu et al. proposed an in situ self-assembly strategy, in which a self-assembled monolayer (SAM) material was introduced into the single-crystal precursor solution so that the hole transport layer nucleated and aligned synchronously with crystal growth (Figure 14c). This in situ integration mode eliminated interfacial voids between the single crystal and the substrate, establishing an atomically intimate charge extraction pathway (Figure 14c) [84]. The study showed that this strategy significantly optimized interfacial energy level alignment, reduced the charge extraction resistance by about 40%, and induced a stronger interfacial built-in electric field. Together, the concepts of stepwise surface defect passivation and in situ self-assembly for lowering the interfacial contact resistance provide an instructive route toward overcoming the charge extraction bottleneck during lateral collection in BC solar cells.

### 4.2. Stability Bottleneck: Electric Field Distribution and Ion Migration Regulation

In principle, the BC architecture possesses excellent operational stability. Li et al. directly compared BC and vertical single-crystal PSCs and found that interfacial iodide ion migration was markedly weaker in the BC configuration, because the ion migration pathway at the metal/perovskite interface is nearly orthogonal to the internal electric field (Table 1) [33].

However, as the thickness of single-crystal films increases, the electric field distribution inside the device becomes increasingly difficult to maintain in an ideal lateral orientation. Under light, heat, or applied bias, the driving force for ion migration is then enhanced, more readily triggering reactions between iodide ions and the electrodes. Jacobs et al. showed that lateral potential differences induced by electrode edges can drive in-plane ion migration and accelerate degradation in halide perovskite devices. As a result, electrochemical degradation can occur at the electrode edges, which accelerates interfacial deterioration and ultimately leads to pronounced decay in long-term operational performance [87].

#### Optimization Pathway: Dimensional Confinement and Hybrid Structure Strategies

**Figure 15:**
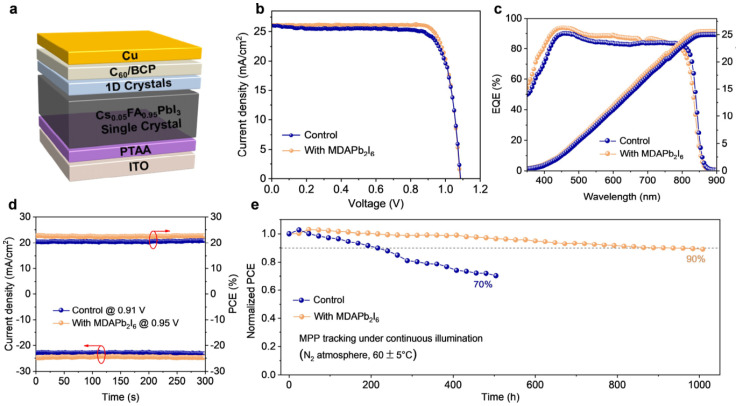
(**a**) Device configuration of single-crystal PSCs. (**b**) *J*–*V* curves of single-crystal PSCs with and without MDAPb_2_I_6_ modification under AM 1.5 light illumination. (**c**) EQE curves and integrated *J*_SC_ of Cs_0.05_FA_0.95_PbI_3_ devices with and without MDAPb_2_I_6_ modification. (**d**) Steady output efficiency and current density at the maximum power point. (**e**) PCE operational stability test of Cs_0.05_FA_0.95_PbI_3_ single-crystal PSCs with and without MDAPb_2_I_6_ modification [88]. Copyright 2024, Wiley-VCH.

The incorporation of low-dimensional perovskite materials is an important route toward improved stability. The physical confinement effect of long-chain organic cations in low-dimensional materials can effectively raise the exciton binding energy while constructing ion migration barriers. To address the high density of trap states and severe ion migration at single-crystal surfaces, Jiang et al. proposed a strategy for the in situ growth of a one-dimensional (1D) perovskite-blocking layer. By constructing a 1D MDAPb_2_I_6_ layer (MDA = 4,4′-methylenedianiline) on the surface of Cs_0.05_FA_0.95_PbI_3_ single crystals, they utilized the very high ion migration activation energy of the 1D phase to build a robust ion-blocking layer (Figure 15a). This 1D/3D heterostructure not only effectively filled surface iodide vacancies and suppressed nonradiative recombination, but it also physically blocked both lateral and vertical penetration of iodide ions toward the electrodes. Photovoltaic characterizations confirm that the *J*–*V* curves (Figure 15b) reveal comparable performance between MDAPb_2_I_6_-modified and control single-crystal PSCs under AM 1.5G illumination. The EQE spectra and integrated *J*_SC_ (Figure 6c) verify efficient light harvesting and charge collection in the modified device. Furthermore, steady-state MPP tracking (Figure 15d) demonstrates stable power output for both devices, with the MDAPb_2_I_6_-modified device operating at a higher voltage of 0.95 V.

Experimental results showed that the single-crystal solar cells modified with the 1D blocking layer exhibited excellent stability under continuous illumination at 60 °C, with a T90 lifetime of up to 1000 h (Figure 15e). Introducing low-dimensional phases at the surface or interface of BC single crystals can therefore synergistically combine the superior transport properties of single crystals with the higher stability of low-dimensional materials, offering a route that simultaneously enhanced efficiency and prolonged operational stability [88].

### 4.3. Large-Area Fabrication Bottleneck: The Conflict Between Scale-Up and Crystallization Uniformity

At present, high-efficiency BC PSCs still rely heavily on small crystals and locally optimized electrode structures; the controllable fabrication of large-area highly crystalline single-crystal films remain a key challenge for scalable BC devices. Moazzezi et al. found that, in the space-confined method, single-crystal film scale-up is mainly limited by restricted mass transport within the narrow confined gap. As the lateral size increases, the precursor supply over long distances becomes increasingly difficult, which can disturb the local supersaturation environment and lead to undesired nucleation, imbalanced growth, and defect formation [59]. Liu et al. identified mass transfer as a major limiting factor in the solution growth of perovskite thin monocrystals, demonstrating that fast mass transfer can ensure uniform precursor supply and suppress bulk defect formation [89]. These results highlight the importance of continuous solute supply for large-area single-crystal wafer growth. For BC devices, the resulting nonuniform crystallization and defect formation would deteriorate long-range lateral carrier transport and charge collection, ultimately limiting the efficiency of large-area devices.

#### Optimization Pathway: Continuous Mass Transfer and Epitaxial Integration

Practical application of BC single-crystal PSCs requires the simultaneous solution of two key issues: the scalable and controllable growth of high-quality perovskite single-crystal wafers, and their efficient integration with patterned back-contacted electrodes. The former requires continuous solute transport and uniform crystallization regulation during crystal growth, whereas the latter relies on high-resolution electrode patterning and efficient lateral carrier collection.

The first challenge lies in overcoming the mass transport limitation during confined single-crystal growth. By increasing the solute flux to 1.65 × 10^−5^ g mm^−2^ min^−1^, Moazzezi et al. enlarged the crystal size by approximately four times, reaching 19 mm^2^, and achieved a growth velocity of up to 34 μm min^−1^ [59]. Liu et al. further identified mass transfer as a major limiting factor in the solution growth of perovskite thin monocrystals and developed a high solute flux strategy to accelerate crystal growth. As a result, the mass diffusion coefficient was increased from 1.7 × 10^−10^ to 5.4 × 10^−10^ m^2^ s^−1^, enabling the growth of 29 types of perovskites thin monocrystals at 40–90 °C with a maximum growth velocity of 27.2 μm min^−1^. With this strategy, MAPbI_3_ thin monocrystals, with lateral lengths approaching 2.0 cm, could be obtained within one 48 h growth cycle, while the thickness could be tuned from 1 to 60 μm [89].

Once continuous wafer growth is achieved, another key issue is how to integrate the single-crystal film with device substrates without sacrificing crystallinity. Lei et al. reported a solution based epitaxial growth and transfer process, by which the thickness of perovskite single-crystal films could be controlled from approximately 600 nm to 100 μm, while continuous single-crystal films with areas up to 5.5 cm × 5.5 cm were obtained. Compared with directly growing single crystals on device substrates, this method separates crystal growth from device integration and allows the as-grown single-crystal films to be transferred onto target substrates. This transferability is particularly attractive for BC architectures, because it allows single-crystal wafers to be integrated with prepatterned back-contacted electrodes without relying on transparent conductive substrates [68].

In addition to continuous wafer growth, position-controlled nucleation provides another route for scalable and reproducible single-crystal fabrication. Printing-assisted strategies can regulate crystal nucleation and growth through predefined seeds, droplets, or patterned regions, thereby overcoming the randomness of conventional solution growth. Gu et al. reported a seed-printing method, in which preprinted perovskite seeds guide the growth of single-crystal films at designated positions. This approach enables the fabrication of millimeter-sized perovskite single-crystal films with controllable thicknesses and high yields, and the obtained single-crystal films can also be transferred onto different substrates [90]. Furthermore, Gu et al. developed an inkjet-printing method to directly deposit perovskite precursor ink at target locations, enabling direct writing and large-area patterning of perovskite single-crystalline microplate arrays. This lithography-free strategy is suitable for constructing regularly arranged single-crystal optoelectronic device arrays [91].

After large-area wafer fabrication, controllable patterning of back-contacted electrodes becomes the next prerequisite for scalable BC devices. Electrode geometry becomes increasingly important when BC devices are scaled from local electrodes to large-area wafers, because it directly determines the lateral carrier transport distance and charge collection efficiency. Currently, shadow mask-defined sequential electrode deposition is commonly used in lateral or BC single-crystal PSCs. For example, Song et al. defined electrode regions using shadow masks and sequentially deposited the anode, charge transport layers, and cathode, achieving lateral single-crystal PSCs with an electrode spacing of approximately 50 μm. This method is simple and avoids complicated photolithography, making it suitable for laboratory-scale device fabrication [37]. However, although this spacing can be tolerated by high-quality single crystals with long carrier diffusion lengths, it becomes unfavorable for large-area devices as carriers generated far from the electrodes are more likely to recombine before extraction.

To shorten the lateral carrier transport distance, high-resolution and scalable electrode patterning techniques are required. Harwell et al. reported a nanoimprint lithography strategy for fabricating honeycomb-shaped interdigitated back-contacted (IBC) electrodes, with feature widths down to 230 nm. This result indicates that nanoimprint lithography can serve as an advanced alternative to conventional mask deposition or photolithography, offering a promising route for large-area fabrication of high-resolution back-contacted electrodes with reduced spacing [92]. In patterned BC PSCs, Deng et al. further showed that microsphere lithography can fabricate periodic back-contacted electrodes over a large area, achieving a stabilized PCE of 8.6% for a 0.015 cm^2^ device and 2.44% for a 0.75 cm^2^ device [93].

In parallel with patterning resolution, electrode geometry must also be optimized to match the increasing crystal size. IBC electrodes provide a more effective geometry because repeated cathode and anode fingers can divide the single-crystal surface into multiple local collection units, thereby reducing the maximum lateral transport distance and increasing the effective collection area. Park et al. used an interdigitated hierarchical electrode design in lateral single-crystal perovskite solar cells and reduced the electrode spacing to 1.5 μm, which suppressed series resistance and recombination loss during lateral transport and yielded a PCE of 9.50% under 0.1 sun illumination [76]. Lin et al. constructed interdigitated back-contacted PSCs and introduced a mesoporous TiO_2_ charge transport layer into the electrode region, converting the planar electrode into a mesoscopic charge collection interface with 3D contact characteristics. This structure significantly increased the interfacial area between the perovskite and the electron selective contact layer, shortened the charge transfer lifetime, and balanced electron and hole extraction, ultimately yielding a short-circuit current density of 21.3 mA cm^−2^ in IBC devices [94]. These results indicate that reducing electrode spacing, increasing effective contact area, and balancing cathode/anode collection are critical for improving lateral charge collection in large-area BC PSCs.

From an industrial perspective, the conflict between scale-up and crystallization uniformity is a central issue limiting the fabrication of high-efficiency perovskite modules. Gao et al. showed that scalable all-perovskite tandem modules are strongly limited by nonuniform crystallization of blade-coated Pb-Sn perovskite layers and defect-rich buried interfaces. By introducing aminoacetamide hydrochloride (AAMCl) to homogenize crystallization, extend the processing window, and selectively passivate buried interfacial defects, they fabricated 40 all-perovskite tandem modules with an average efficiency of 23.3 ± 0.7%. A 20.25 cm^2^ aperture area module achieved a certified stabilized efficiency of 24.5% with a geometric fill factor of 96.1%, corresponding to an active area efficiency of 25.9% [95]. Xie et al. recently addressed this issue from a module manufacturing perspective by investigating the crystallization nonuniformity and local damage generated around laser-scribed interconnection regions. Although such effects are usually negligible in small-area cells, they can directly influence both efficiency and stability in modules. The authors found that the regions near the laser-scribed lines exhibited inferior crystallization quality and were more susceptible to degradation, while subsequent laser-processing steps could introduce localized thermal damage and induce perovskite decomposition. By introducing (E)-but-2-ene-1,4-diamine dihydrochloride (BDECl) to regulate the crystallization kinetics of perovskites near the scribed regions, they improved film crystallinity and environmental tolerance. As a result, modules with aperture areas of 25 and 100 cm^2^ achieved efficiencies of 24.70% and 23.89%, respectively, with the 100 cm^2^ module further obtaining a certified efficiency of 23.55%. Moreover, the unencapsulated modules retained 93% of their initial efficiency after 3120 h of storage in ambient air under the ISOS-D-1 protocol [96].

## 5. Conclusions and Outlook

**Figure 16:**
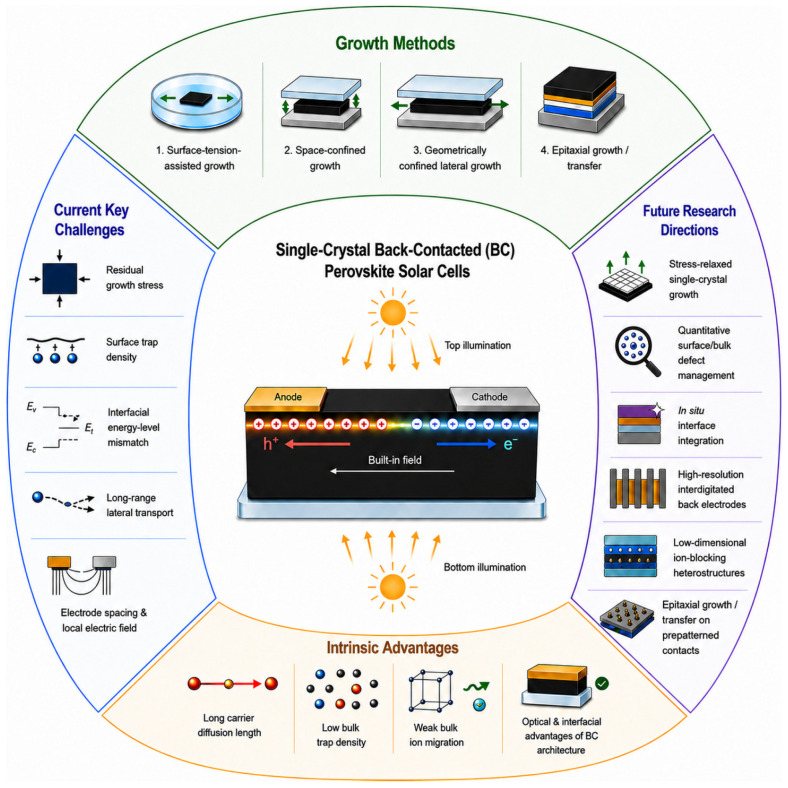
Summary of key challenges and future optimization pathways for single-crystal BC PSCs.

In summary, single-crystal BC PSCs represent a promising but still immature photovoltaic architecture, combining the long carrier diffusion length, low bulk trap density, and weak bulk ion migration of perovskite single crystals with the optical and interfacial advantages of back-contacted devices. Recent progress has increased the efficiency of these devices from early proof-of-concept values to over 17%, with operational stability currently extended to more than 1300 h. Nevertheless, their long-term operational stability still lags significantly behind that of commercial single-crystal silicon solar cells. This is mainly because charge collection in BC devices is governed by long-range lateral transport, which is highly sensitive to residual growth stress, surface trap density, interfacial energy level mismatch, electrode spacing, and local electric field distribution. Accordingly, further stability improvement can be achieved via crystal engineering, surface defect passivation, stress regulation, and construction of low-dimensional perovskite structures to suppress ion migration. More critically, future development cannot rely only on small-area champion devices; the key challenge is to simultaneously achieve wafer-scale crystallization uniformity, low defect density, controllable thickness, robust interfacial contact, and scalable electrode patterning. Therefore, future research should focus on stress-relaxed single-crystal growth, quantitative surface and bulk defect management, in situ interface integration, high-resolution interdigitated back-contacted electrodes, low-dimensional ion-blocking heterostructures, and epitaxial growth or transfer strategies compatible with prepatterned contacts (Figure 16). At the same time, industrially relevant metrics, including cell-to-module efficiency loss, long-term operational stability, and energy payback time, should be incorporated into the evaluation of large-area BC single-crystal devices. With coordinated advances in crystal growth, interfacial physics, device geometry, and scalable manufacturing, single-crystal BC perovskite solar cells may evolve from a laboratory-scale model system into a viable route toward stable, low-cost, and high-efficiency perovskite photovoltaics.

## Figures and Tables

**Table 1:** Comparison of representative unencapsulated vertical and BC single-crystal PSCs under MPP tracking.

Architecture	Device Structure	Stability	Ref.
Vertical structure	ITO/PTAA:P3HT/MAPbI_3_/C_60_/BCP/Cu	T100 ≈ 200 h	[85]
Vertical structure	ITO/PTAA/FA_0.55_MA_0.45_PbI_3_/C_60_/BCP/Cu	T100 = 330 h	[86]
Vertical structure	ITO/MeO-2PACz/FA_0.6_MA_0.4_PbI_3_/C_60_/BCP/Cu	T100 ≈ 960 h	[84]
BC structure	Au/MAPbI_3_/C_60_/BCP/Au	T100 = 200 h	[37]
BC structure	Au/FA_0.75_MA_0.25_PbI_3_/C_60_/BCP/Au	T100 = 1200 h	[33]
BC structure	Au/MoO_3_/Cs_0.05_FA_0.95_PbI_3_/C_60_/BCP/Cu	T95 > 1560 h	[41]

## Data Availability

No new data were created or analyzed in this study. Data sharing is not applicable to this article.

## Abbreviations

The following abbreviations are used in this manuscript:

BC	Back-contacted
ITO	Indium tin oxide
FTO	Fluorine-doped tin oxide
XRD	X-ray diffraction
SEM	Scanning electron microscope
AFM	Atomic force microscopy
IBC	Interdigitated back-contacted
PSC	Perovskite solar cell
